# Mediterranean Diet: What Are the Consequences for Menopause?

**DOI:** 10.3389/fendo.2022.886824

**Published:** 2022-04-25

**Authors:** Claudia Vetrani, Luigi Barrea, Rosa Rispoli, Ludovica Verde, Giulia De Alteriis, Annamaria Docimo, Renata Simona Auriemma, Annamaria Colao, Silvia Savastano, Giovanna Muscogiuri

**Affiliations:** ^1^ Department of Clinical Medicine and Surgery, Endocrinology Unit, University of Naples “Federico II”, Naples, Italy; ^2^ Dipartimento di Scienze Umanistiche, Università Telematica Pegaso, Naples, Italy; ^3^ Centro Italiano per la cura e il Benessere del Paziente con Obesità (C.I.B.O), Department of Clinical Medicine and Surgery, Endocrinology Unit, Federico II University Medical School of Naples, Naples, Italy; ^4^ UNESCO Chair “Education for Health and Sustainable Development”, University of Naples “Federico II”, Naples, Italy

**Keywords:** menopause, menopausal symptoms, Mediterranean diet, sleep quality, PREDIMED score, MRS

## Abstract

Menopause is a natural event occurring in a woman’s life that is often accompanied by symptoms that might affect the quality of life. Diet has been shown to influence menopausal-related symptoms. Therefore, the present study aimed to investigate whether the adherence to the Mediterranean Diet (MD) might influence menopausal symptoms in women with obesity. This cross-sectional study involved postmenopausal women with obesity. Anthropometric and clinical parameters, and lifestyle habits were evaluated. All participants underwent interview questionnaires to assess: the adherence to the MD (PREDI PREvencion con DIetaMEDiterranea, PREDIMED), sleep quality (Pittsburgh Sleep Quality Index, PSQI), and severity of menopausal symptoms (Menopausal Rating Scale, MRS). One hundred postmenopausal women were enrolled (age 57.1 ± 7.3 years, BMI 35.0 ± 5.5 kg/m^2^). The mean PREDIMED score was 7.82 ± 1.66 showing moderate adherence to MD. Women in the marked MRS class had a significantly lower PREDIMED score than the none-to-moderate MRS class (p=0.036). The intake of legumes was associated with a lower MRS class (r= -0.201, p=0.045). In addition, the intake of extra-virgin olive oil inversely correlated with psychological symptoms (r= -0.230 p=0.021). Finally, 79% of participants were poor sleepers (mean PSQI score was 8.68 ± 3.6) and women in the severe MRS class had a worse sleep quality compared to other MRS classes. Post-menopausal women with marked menopausal symptoms had low adherence to MD. Legume consumption was associated with lower menopausal symptoms severity while extra virgin olive oil consumption was associated with lower psychological symptoms.

## 1 Introduction

Menopause is the physiological cessation of reproductive capacity in a woman’s life. Usually, it occurs between 45-55 years unless pathological conditions induce premature or early menopause (1).

Menopausal transition (also known as climacteric) starts with the onset of menstrual abnormalities and ends with the last menstrual period. Physiological changes during climacteric -mainly related to hormonal variations - may trigger a variety of menopausal symptoms that might last years in the postmenopausal period (2, 3).

Menopausal symptoms include psychological (depressive mood, irritability, anxiety, tiredness), somato-vegetative (hot flushes, palpitations, insomnia, muscle and joint pain), and urogenital (sexual problems, bladder problems, and vaginal dryness) disorders (1–3). Notably, several cohort studies have demonstrated an association between menopausal symptoms and lower quality of life (4–8).

In addition, hormonal changes during menopause contribute significantly to weight gain, increased visceral fat mass, and the development of abdominal obesity (3). Therefore, postmenopausal women may also present obesity-related metabolic disorders (i.e., insulin-resistance, dyslipidemia, and metabolic syndrome) that associate with an increased risk of type 2 diabetes, cardiovascular disease, and other chronic-degenerative diseases (9–11).

The Mediterranean diet (MD) is a healthy dietary pattern that has been associated with a reduced risk of cardiovascular events, morbidity and overall mortality, and the improvement of body weight, metabolic profile, and cognitive function (11–14).

MD is characterized by a wide variety of plant foods, such as vegetables, fruits, legumes, nuts, and whole-grain cereals, and the use of extra-virgin olive oil as the main source of fat (15). Therefore, MD provides low amounts of saturated fats in favor of unsaturated fats, and many bioactive compounds with anti- inflammatory and antioxidant activities (omega 3 fatty acids and vitamins, minerals, and phytochemicals, respectively) (15). More in detail, most of MD energy intake comes from carbohydrate (55%), close to 30% from fat, and the remaining part by protein (15%). Fats are mainly represented by 19% of monounsaturated fatty acids (MUFA), followed by saturated fatty acids (SFA, 9%) and polyunsaturated fatty acids (PUFA, 5%), and cholesterol is 300 mg/day (15).

Several studies suggested that MD might play a role in the reduction of body weight and menopausal symptoms in postmenopausal women by virtue of its dietary composition. In particular, a cross-sectional study in 481 postmenopausal women showed that a high adherence to the MD, evaluated by the Mediterranean Diet Score (MDS), was negatively associated with body weight, waist circumference, and waist-to-height ratio (16). Moreover, a cross-sectional study in 8.954 Spanish perimenopausal-post-menopausal women showed that high adherence to the MD was associated with a lower prevalence and risk of being overweight/obese (17).

As for menopausal symptoms, Herber-Gast and colleagues (18) investigated the relationship between MD and risk of vasomotor symptoms (hot flushes and night sweats) in the Australian Longitudinal Study on Women’s Health, a cohort study involving 6,040 postmenopausal women with a 9 year- follow-up. In this study a higher adherence to MD was inversely associated with vasomotor menopausal symptoms, thus suggesting that MD might be useful to prevent vasomotor menopausal symptoms.

Against this background, the aim of the present study was to investigate the impact of adherence to MD on the severity of menopausal symptoms in postmenopausal women with obesity. In addition, we explored the possible association between postmenopausal symptoms and the main foodstuffs characterizing MD.

## 2 Methods

### 2.1 Study Population

All postmenopausal women with obesity attending the Outpatient Clinic of the Unit of Endocrinology, Department of Clinical Medicine and Surgery, University of Naples “Federico II” from May 2021 to October 2021 were screened for eligibility. The inclusion criteria were menopause status established after 1 year since the last menstrual period (amenorrhea for at least 12 months in the absence of treatment with hormonal contraceptives, or hysterectomized women with menopausal symptoms) and obesity (BMI >30 kg/m^2^). The exclusion criteria were the following: normal weight or overweight (BMI< 30 kg/m^2^), premenopausal status, hormonal therapy, type 1 diabetes mellitus, women with diabetes on insulin therapy, any other chronic disease (cardiopulmonary, brain, kidney, or liver disease), severe mental illness (depression, anxiety, or schizophrenia), women with allergy/intolerance or following a specific dietary regimen.

The aim of the study was clearly explained to all potential participants and written informed consent was obtained. The study was approved by the Local Ethical Committee and carried out in accordance with the Declaration of Helsinki.

### 2.2 Study Design

This cross-sectional study was conducted in compliance with the Strengthening the Reporting of Observational Studies in Epidemiology (STROBE) statement checklist (19).

Trained medical doctors and nutritionists collected demographic information, medical history, lifestyle habits (smoke, alcohol use, physical activity), and anthropometric parameters. All participants underwent interview questionnaires to assess: adherence to MD (PREDI PREvencion con DIetaMEDiterranea, PREDIMED) (20), sleep quality (Pittsburgh Sleep Quality Index, PSQI) (21), and severity of menopausal symptoms (Menopausal Rating Scale, MRS) (22).

#### 2.2.1 Demographic Information, Medical History, Lifestyle Habits

A brief medical history was used to assess the presence of diseases (type 2 diabetes, cardiovascular diseases, hypertension, dyslipidemia) and pharmacological therapy. In addition, information about age at menarche, years since menopause, type of menopause (natural or induced), weight before menopause, weight gain after menopause, and full-term pregnancies were collected.

As for lifestyle habits, smoking at least one cigarette/day identified “current smokers”. Participants habitually engaged in at least 30 minutes/day of aerobic exercise were defined as physically active. Alcohol use was identified with a threshold of more than 20 g/day.

#### 2.2.2 Anthropometric Measurements

Anthropometric measurements were performed by the same operator (a nutritionist experienced in providing nutritional assessment and body composition), according to standard procedures (23, 24).

All measurements (weight, height, waist and hip circumferences) were performed on participants wearing light clothing and no shoes, after an overnight fast, according to standard procedures (the subject was standing upright with the feet together and the arms hanging loosely by the sides, with the subject standing and breathing normally, as previously reported (25–27). Waist (WC) and hip circumferences (HC) were used to calculate the waist-hip ratio (WHR). Weight and height were used to calculate the body mass index (BMI). Obesity was classified according to World Health Organization’s criteria (28): Obesity grade I (BMI: 30.0-34.9 kg/m^2^); Obesity grade II (BMI: 35.0-39.9 kg/m^2^); Obesity grade III (BMI >40.0 kg/m^2^).

#### 2.2.3 Interview Questionnaires

The adherence to MD was assessed using the PREDIMED questionnaire, consisting in 14 items (intake and amount of extra-virgin olive oil, frequency of fruit, vegetables, nuts, legumes, red meat, poultry, fish, animal fat, sweetened beverages, sweets, and sofrito). A qualified nutritionist administered this questionnaire with the same face-to-face interview used in previous studies (29). PREDIMED score was calculated by assigning a score of 1 and 0 for each item. According to the PREDIMED score, participants were classified as follows: 0–5, lowest adherence; score 6–9, average adherence; and score ≥10, highest adherence (20).

Overall sleep quality was assessed with the Pittsburgh Sleep Quality Index (PSQI). Poor sleepers were defined as participants with PSQI score ≥ 5 whereas good sleepers were defined as participants with PSQI score < 5 (21).

The Menopausal Rating Scale (MRS) consisted in a list of 11 symptoms (22). The respondents had a choice of score between 0 (no symptoms) and 4 points (severe symptoms), depending on subjective perception. Three groups of symptoms can be detected with MRS: psychological (depression, irritability, anxiety, physical and mental exhaustion), somato-vegetative (sweating and hot flushes, cardiac complaints, sleeping disorders, joint and muscle complaints), and urogenital symptoms (sexual problems, urinary complaints, vaginal dryness). Therefore, the total MRS score ranged between 0 (asymptomatic) and 44 (highest degree of complaints). According to the MRS score, five categories can be identified: no symptoms (0-4), mild (5-8), moderate (9-12), marked (13-16), and severe (>17). Nevertheless, previous studies (30, 31) reported that respondents might have difficulties in rating the perceptions of low-to-moderate symptoms. Therefore, since our primary aim was to investigate the impact of MD on the severity of menopausal symptoms, the classes of “no symptoms”, “mild”, and “moderate” were combined in a single class (“none-to-moderate symptoms”).

#### 2.2.4 Sample Size Justification and Power

The calculation of the sample size was performed *a priori* by considering the effect size 0.3 with a type I error of 0.05 and a power of 80%. The number of subjects to be enrolled was found to be 82. During the study, 100 women were considered eligible for the study. This sample size met the at least necessary number of subjects and would further support the result. Therefore, we decided to include all subjects in the statistical analysis. The calculation of the Sample Size was performed using G Power Software.

#### 2.2.5 Statistical Analysis

Continuous variables were expressed as mean ± standard deviation (SD) whereas categorical variables were reported as numbers and percentages (%). Differences between groups were analyzed by analysis of variance (one-way ANOVA) and *post hoc* analyses (least significant difference test, LSD). Chi-squared test for independence was used to assess the association between outcomes and categorical variables. Correlations between study variables were performed using Spearman rank correlation. A *p* value <0.05 was considered significant. Statistical analysis was performed according to standard methods using the Statistical Package for Social Sciences software 26.0 (SPSS/PC; SPSS, Chicago, IL, USA).

## 3 Results

A total of 100 postmenopausal women with obesity (BMI 35.0 ± 5.5 kg/m^2^, mean age 57.2 ± 7.3) were included in the analyses. The main characteristics of the study participants (demographic, anthropometric and medical data, lifestyle habits) and the mean score of the interview questionnaires (PREDIMED and PSQI) were reported in **Table 1**.

**Table 1:** Demographic, anthropometric and medical data, lifestyle habits, adherence to the Mediterranean diet, and sleep quality of all study participants.

Parameters	All participants (n= 100)
Age (years)	57.2 ± 7.3
BMI (Kg/m^2^)	35.0 ± 5.5
Grade I obesity	65 (65%)
Grade II obesity	18 (18%)
Grade III obesity	17 (17%)
WC (cm)	107 ± 13
HC (cm)	120 ± 15
WHR	0.89 ± 0.1
Age at menarche (years)	11.8 ± 1.6
Age at menopause (years)	49.0 ± 4.8
Weight before menopause (kg)	81.8 ± 19
Weight gain after menopause (kg)	10.1 ± 14
Years since menopause	8.94 ± 8.3
Full-term pregnancies	87 (87%)
Menopause type	
Natural menopause	87 (87%)
Induced menopause	13 (13%)
Diseases	
Type 2 diabetes	8 (8%)
Hypertension	39 (39%)
Dyslipidemia	39 (39%)
Medications	
Antihypertensive	36 (36%)
Glucose-lowering drugs	6 (6%)
Statins	8 (8%)
Anticoagulant	7 (7%)
Smoking	23 (23%)
Alcohol	12 (12%)
Physical activity	21 (21%)
PREDIMED score	7.82 ± 1.7
Low adherence (0–5)	8 (8%)
Average adherence (6–9)	77 (77%)
High adherence (≥10)	15 (15%)
PSQI	8.68 ± 3.6
Good Sleepers (≥ 5)	21 (21%)
Poor Sleepers (< 5)	79 (79%)

Data are expressed as mean ± SD or n (%).

BMI, body mass index; WC, waist circumference; HC, hip circumference; WHR, waist-to-hip-ratio; PREDIMED, adherence to the Mediterranean diet (Prevención con Dieta Mediterránea); PSQI, Pittsburgh Sleep Quality Index.

Seventy-five (65%) participants presented grade I obesity whereas women with grade II and grade III were 18 (18%) and 17 (17%), respectively.

WC was 107.4 ± 13 cm, HC was 120.3 ± 14.8, and WHR was 0.89 ± 0.06. As for lifestyle habits, 23 women (23%) were smokers, and most of the participants were sedentary 79 (79%). The prevalence of diseases was: 8% type 2 diabetes, 39% hypertension, and 39% dyslipidemia. Participants taking medications were 36% antihypertensive, 6% antidiabetic, 8% hypolipidemic medications, and 7% anticoagulant medications.

The mean PREDIMED score was 7.82 ± 1.7. Eight (8%) participants presented low adherence, 77 (77%) had average adherence, while 15 (15%) had high adherence. The mean PSQI score was 8.68 ± 3.6. Twenty-one participants (21%) were good sleepers, while 79 (79%) were poor sleepers.

Total MRS scores and the severity of symptoms are reported in **Table 2**. The mean MRS score was 22.5 ± 8.5, with the following prevalence of the severity of symptoms: 11 women (11%) none-to-moderate symptoms, 12 (12%) marked symptoms, and 77 (77%) severe symptoms. Regarding the somatic and psychological symptoms, most of the participants were in the third class of symptoms severity (39% and 29%, respectively), whereas 52% of participants were in the first class of severity for urogenital symptoms. As for the specific menopausal symptom, the most frequent disorders were sleep problems (80%), depressive mood (79%), anxiety (79%), physical and mental exhaustion (79%), and joint and muscular discomfort (79%).

**Table 2:** Frequency distribution of Menopause Rating Scale (MRS) scores and menopausal symptoms.

MRS and symptoms	All participants (n= 100)
**Total MRS score**	22.5 ± 8.5
- None-to-moderate symptoms (0-12)	11 (11%)
- Marked (13-16)	12 (12%)
- Severe (17+)	77 (77%)
**Somatic score**	8.50 ± 4.1
0-4	18 (18%)
5-8	27 (27%)
9-12	39 (39%)
13-16	16 (16%)
**Psychological score**	8.89 ± 4.4
0-2	14 (14%)
5-8	35 (35%)
9-12	29 (29%)
13-16	22 (22%)
**Urogenital score**	5.02 ± 3.2
0-4	52 (52%)
5-8	32 (32%)
9-12	16 (16%)
**Symptoms**	
Hot flashes and sweating	74 (74%)
Heart discomfort	59 (59%)
Sleep problems	80 (80%)
Depressive mood	79 (79%)
Irritability	76 (76%)
Anxiety	79 (79%)
Physical and mental exhaustion	79 (79%)
Sexual problems	75 (75%)
Bladder problems	42 (42%)
Dryness of the vagina	62 (62%)
Joint and muscular discomfort	79 (79%)

Data are expressed as mean ± SD or n (%).

**Table 3** reported the population’s characteristics stratified by severity of menopausal symptoms identified according to the MRS score. No significant differences were observed for age, BMI, obesity grade, other anthropometric measures (WC, HC, and WHR), lifestyle habits (smoking, alcohol, physical activity), and medical history (prevalence of diseases, medications, and menopause-related data). However, a significantly lower PREDIMED score we observed in the “marked symptoms” class than the “none-to-moderate symptoms” class (7.08 ± 1.2 *vs*. 9.50 ± 0.7, respectively; p=0.036). The “severe symptoms” class presented a lower PREDIMED score than the “none-to-moderate symptoms” class (7.83 ± 1.6 *vs*. 9.50 ± 0.7, respectively; p=0.181), although the difference did not reach the conventional level of statistical significance.

**Table 3:** Demographic, anthropometric and medical, lifestyle habits, adherence to the Mediterranean diet, and sleep quality of participants stratified by severity of menopausal symptoms (MRS).

Parameters	None-to moderate symptoms (n=11)	Marked Symptoms (n=12)	Severe Symptoms (n=77)	p-value*
Age (years)	61.0 ± 5.3	57.8 ± 8.5	56.5 ± 7.3	0.143
BMI (Kg/m^2^)	33.5 ± 4.4	34.0 ± 3.5	35.4 ± 5.8	0.450
Grade I obesity	8 (73%)	8 (67%)	49 (64%)	0.833
Grade II obesity	2 (18%)	3 (25%)	13 (17%)	0.793
Grade III obesity	1 (9%)	1 (8%)	15 (19%)	0.481
WC (cm)	105 ± 11	102 ± 8.9	109 ± 14	0.458
HC (cm)	114 ± 9.3	124 ± 16	124 ± 16	0.102
WHR	0.93 ± 0.10	0.90 ± 0.04	0.88 ± 0.05	0.208
Age at menarche (years)	12.2 ± 1.3	11.8 ± 1.4	11.7 ± 1.6	0.674
Age at menopause (years)	49.2 ± 4.4	49.5 ± 5.5	48.8 ± 4.7	0.896
Weight before menopause (kg)	76.6 ± 20	78.9 ± 12	82.9 ± 18	0.462
Weight gain after menopause (kg)	10.9 ± 13	12.9 ± 6.1	9.58 ± 15	0.734
Years since menopause	12.2 ± 6.5	9.67 ± 7.3	8.36 ± 8.7	0.350
Full-term pregnancies	2 (18%)	10 (83%)	67 (87%)	0.812
Menopause type				
Natural menopause	10 (91%)	10 (83%)	67 (87%)	0.864
Induced menopause	1 (9%)	2 (17%)	10 (13%)	
Diseases				
Type 2 diabetes	0 (0%)	0 (0%)	8 (10%)	0.273
Hypertension	5 (45%)	5 (42%)	29 (38%)	0.867
Dyslipidemia	6 (54%)	4 (33%)	29 (38%)	0.512
Medications:				
Antihypertensive	2 (18%)	6 (50%)	28 (36%)	0.281
Glucose-lowering drugs	2 (18%)	0 (0%)	4 (5%)	0.153
Statins	1 (1%)	0 (0%)	7 (9%)	0.553
Anticoagulant	2 (18%)	1 (8%)	4 (5%)	0.282
Smoking	2 (18%)	4 (33%)	17 (22%)	0.636
Alcohol intake	3 (27%)	1 (8%)	8 (10%)	0.250
Physical activity	2 (18%)	1 (8%)	18 (23%)	0.478
PREDIMED score	9.50 ± 0.7	7.08 ± 1.2^a^	7.83 ± 1.6	0.108
PSQI score	4.00 ± 1.4^b^	6.08 ± 2.9^b^	9.39 ± 3.5	0.001

Data are expressed as mean ± SD or n (%).

BMI, body mass index;WC, waist circumference; HC, hip circumference; WHR, waist-to-hip-ratio; PREDIMED, adherence to the Mediterranean diet (Prevención con Dieta Mediterránea); PSQI, Pittsburgh Sleep Quality Index.

*one-way ANOVA or chi-square test. ^a^p < 0.05 vs. “none-to moderate symptoms” class, one-way ANOVA and at least difference (LSD) post hoc test for multiple comparisons. ^b^p< 0.05 vs. “severe symptoms” class, one-way ANOVA and at least difference (LSD) post hoc test for multiple comparisons.

PSQI score was significantly lower in the “severe symptoms” class than in “marked symptoms” (p=0.012) and “none-to-moderate symptoms” (p=0.002) classes.

Correlation analysis was performed to assess the association of menopausal symptoms with PREDIMED items. The intake of legumes (≥3 times/week) was inversely correlated to the MRS score (r= -0.201, p=0.045). The intake of extra-virgin olive oil was inversely correlated with psychological symptoms (r: -0.230, p=0.021), mainly related to reduced depressive mood (r= -0.205, p=0.041). No other significant correlations with PREDIMED items and menopausal symptoms were observed.

## 4 Discussion

The present study showed that the severity of menopausal symptoms (assessed by MRS) was inversely associated with the adherence to MD. In particular, women in the marked MRS class had a significantly lower adherence to MD than women in the none-to-moderate MRS class. Therefore, we investigated what food component characterizing MD was mostly involved in this association. The results showed that the intake of legumes and extra-virgin olive oil was associated with lower severity of total menopausal symptoms and psychological symptoms, respectively. Furthermore, women in the severe MRS class presented a lower sleep quality compared to other classes.

The association between menopausal symptoms and adherence to MD has been previously reported in two large cohort studies in postmenopausal women with overweight/obesity. Interestingly, a 2-years clinical trial in demonstrated that postmenopausal women with overweight/obesity (n=116, aged 56.6 ± 3.8 years, BMI 29.6 ± 3.8 kg/m^2^) advised a MD-resembling diet (low intake of meat, pastries, cakes, and sweets, while high intake of virgin olive oil, nuts, vegetables, legumes, and fruits) experienced an improvement of menopausal symptoms (32).

The present study added information about the specific components of MD that might explain the association with post-menopausal symptoms. Although no previous studies focused on the consumption of legumes intake and the severity of menopausal symptoms, two meta-analyses of randomized controlled trials (33, 34) reported an inverse association between post-menopausal symptoms and soy intake - known to be a legume. The mechanism behind this effect seems to be related to the activity of isoflavones. Indeed, isoflavones are biologically active compounds, with a chemical structure resembling estrogens. Therefore, they can bind estrogen-receptors thus positively influencing menopausal symptoms (35, 36). Dietary intake of isoflavones ranges from 5 to 80 mg/day in Asia, whereas in Western populations the reported daily intake is usually less than 3 mg/day (37). However, a study carried out in 14.029 individuals living in Southern Italy (38), with a strong Mediterranean gastronomic background, reported an isoflavone intake ranging 18-31 mg/day.

Regarding the use of extra-virgin olive oil, in this study women who consumed extra virgin olive oil experienced less psychological symptoms, in particular depressive mood.

Although this finding has not been observed in postmenopausal women, a 12-week nutritional trial carried out in 149 individuals with severe obesity (BMI > 35 Kg/m^2^) showed that daily consumption of extra virgin olive oil (52ml/day) significantly reduced depression and anxiety (39). In line with this, Foshati and colleagues demonstrated that the consumption of extra-virgin olive oil (25 ml/day for 7 weeks) significantly improved depressive symptoms in 73 patients with severe depression (40).

Although the psychological implications of diet are not completely clear (41, 42), pro-inflammatory foods such as those rich in saturated fats may induce the release of pro-inflammatory cytokines impairing the activity of the dopaminergic system, while the intake of monounsaturated fatty acids has shown to improve brain function (39).

Finally, as expected, participants in the severe MRS class presented a worsen sleep quality than other MRS classes. This finding is in line with a previous study carried out in 385 postmenopausal women aged <45 years investigating sleep quality by PSQI and its association with symptoms related to the menopausal period. In this study women who presented greater severity of menopausal symptoms reported greater impairment in sleep quality (43). The etiology of sleep disorders in menopausal women is still controversial. Some evidence suggested a role of the reduction of estrogens levels that can induce depression (44) and vasomotor symptoms (45). In addition, weight gain and particularly increased visceral adiposity can influence sleep quality through the secretion of cytokines or indirectly through obstructive sleep apnea that is frequently in individuals with severe obesity (46–48).

Although no correlation was found between sleep disorders and adherence to MD, nutrition might play a role in the modulation of sleep quality. Indeed, the intake of phytoestrogens has been associated with a reduction of the frequency of insomnia (49). Moreover, a high intake of omega-3 has been reported to decrease depression and anxiety which could contribute to improve sleep quality (50).

Nevertheless, we can not exclude the possibility that sleep disorders would worse the menopausal symptoms, at least the psychological aspects.

The present study had both strengths and limitations. It included a relatively large sample of postmenopausal women for exploring associations between MD and menopausal symptoms. However, it was a convenience sample, as participants were consecutively recruited from women attending the Outpatient Clinic of the Unit of Endocrinology, Department of Clinical Medicine and Surgery, University of Naples “Federico II”. In addition, the cross-sectional design of the study did not allow for cause-effect conclusions. Finally, all questionnaires used in this study were translated in Italian from the English version with no forward-backward translation method to guarantee the conceptual equivalence (51). Nevertheless, the participants were interviewed by expert operators thus increasing the reliability of the collected data.

In conclusion, in the present study, the adherence to MD was associated to lower severity of menopausal symptoms in women with obesity. Notably, legume consumption was associated with lower menopausal symptoms severity, and extra virgin olive oil was associated with a reduction in psychological symptoms. These findings highlight the importance of the MD as an ideal nutritional strategy in the management of menopause.

## Data Availability Statement

The raw data supporting the conclusions of this article will be made available by the authors, without undue reservation.

## Ethics Statement

The studies involving human participants were reviewed and approved by University of Naples Federico II Ethics Committee. The patients/participants provided their written informed consent to participate in this study.

## Author Contributions

The authors’ responsibilities were as follows CV, LB, and GM: were responsible for the concept and design of the study, interpreted data and drafted the manuscript. CV, LB, and RR: conducted statistical analyses. RR, LV, GA, AD, and RA: collected data. AC, SS, and GM: provided a critical review of the manuscript. All authors contributed to and approved the final version of the manuscript.

## Conflict of Interest

The authors declare that the research was conducted in the absence of any commercial or financial relationships that could be construed as a potential conflict of interest.

## Publisher’s Note

All claims expressed in this article are solely those of the authors and do not necessarily represent those of their affiliated organizations, or those of the publisher, the editors and the reviewers. Any product that may be evaluated in this article, or claim that may be made by its manufacturer, is not guaranteed or endorsed by the publisher.
